# Transgenic supplementation of SIRT1 fails to alleviate acute loss of nigrostriatal dopamine neurons and gliosis in a mouse model of MPTP-induced parkinsonism

**DOI:** 10.12688/f1000research.6386.1

**Published:** 2015-05-27

**Authors:** Yasuko Kitao, Natsumi Ageta-Ishihara, Ryosuke Takahashi, Makoto Kinoshita, Osamu Hori

**Affiliations:** 1 Department of Neuroanatomy, Kanazawa University, Takara-machi, Kanazawa, 920-8640, Japan; 2 Department of Molecular Biology, Division of Biological Sciences, Nagoya University Graduate School of Science, Furo-cho, Chikusa, Nagoya 464-8602, Japan; 3 Department of Neurology, Kyoto University Graduate School of Medicine, Kyoto, 606-8501, Japan; 4 Core Research for Evolutionary Science and Technology (CREST), Japan Science and Technology Agency (JST), Kawaguchi, Japan

**Keywords:** Dopamine, 1-methyl-4-phenyl-1,2,3,6-tetrahydropyridine (MPTP), resveratrol, SIRT1, Sirtuin

## Abstract

**Background**

Dopamine (DA) neuron-selective uptake and toxicity of 1-methyl-4-phenyl-1,2,3,6-tetrahydropyridine (MPTP) causes parkinsonism in humans. Loss of DA neurons via mitochondrial damage and oxidative stress is reproduced by systemic injection of MPTP in animals, which serves as models of parkinsonism and Parkinson’s disease (PD). This study aimed to test whether pan-neural supplementation of the longevity-related, pleiotropic deacetylase SIRT1, which confers partial tolerance to at least three models of stroke and neurodegeneration, could also alleviate MPTP-induced acute pathological changes in nigrostriatal DA neurons and neighboring glia.

**Results**

We employed a line of prion promoter-driven *Sirt1*-transgenic (Sirt1Tg) mice that chronically overexpress murine SIRT1 in the brain and spinal cord. Sirt1Tg and wild-type (WT) male littermates (3‒4 months old) were subjected to intraperitoneal injection of MPTP. Acute histopathological changes in the midbrain and striatum (caudoputamen) were assessed with serial coronal sections triply labeled for tyrosine hydroxylase (TH), glial fibrillary acidic protein (GFAP), and nuclear DNA. In the substantia nigra pars compacta (SNpc) of the midbrain, the number of TH-positive neurons and the reactive gliosis were comparable between the Sirt1Tg and WT littermates. In the striatum, the relative fluorescence intensity of TH-positive nerve terminals and the level of gliosis did not differ by the genotypes.

**Conclusions**

Sirt1Tg and WT littermate mice exhibited comparable acute histopathological reactions to the systemic injection of MPTP, loss of TH-positive neurons and reactive gliosis. Thus, the genetic supplementation of SIRT1 does not confer histologically recognizable protection on nigrostriatal DA neurons against acute toxicity of MPTP.

## Introduction

Dopamine (DA) transporter-mediated uptake of 1-methyl-4-phenylpyridine (MPP ^+^), an oxidized metabolite of 1-methyl-4-phenyl-1,2,3,6-tetrahydropyridine (MPTP), damage DA neurons by impairing mitochondrial respiratory chain and generating reactive oxygen species ^1^. The DA neuron-selective toxicity is reproduced in animals by systemic administration of MPTP, which serve as models of Parkinson’s disease (PD) ^2^. The neurotoxicity of MPTP is alleviated by pretreating mice with resveratrol (trans-3,5,4'-trihydroxystilbene) or other phytoalexins ^3– 5^. Besides directly suppressing oxidative stress of MPP ^+^ as an antioxidant ^6^, resveratrol modulates cytoprotective signaling molecules and enzymes that include nicotinamide adenine dinucleotide (NAD ^+^)-dependent deacetylase SIRT1 ^7^. Given the pleiotropic cytoprotective potentials of SIRT1 through diverse substrates, resveratrol’s antagonism against MPTP may be mediated at least in part by SIRT1 ^7, 8^. SIRT1 alleviates animal and cellular models of amyotrophic lateral sclerosis (ALS), Huntington’s disease, Alzheimer’s disease, and an α-synuclein model of PD ^9^. On the other hand, transgenic mice that overexpress SIRT1 in a neuronal subset via the neuron-specific enolase (NSE) gene promoter were not resistant to MPTP ^10^.

We have established a distinct line of transgenic mice that overexpress SIRT1 in wider neuronal lineages and additionally in glial and vascular endothelial cells via the murine prion gene promoter (Prp) ^11, 12^. Unlike the NSE-SIRT1 mice, our Prp-SIRT1 mice are resistant to cerebral hypoperfusion by bilateral common carotid artery stenosis, due to vascular dilatation which is potentiated by SIRT1-mediated deacetylation of endothelial nitric oxide synthase (eNOS) ^12^. Further, Prp-SIRT1 mice are resistant to proteotoxic stress by an ALS-linked mutant of superoxide dismutase 1 (SOD1), due partly to SIRT1-mediated deacetylation of the heat shock factor 1 (HSF1) and the resulting upregulation of HSP70i ^11^. On the basis of the SIRT1-HSF1 axis, and the protective effects of HSF1/HSPs against neurodegenerative insults such as MPTP and α-synuclein ^9, 13– 16^, we assessed whether Prp-SIRT1 mice is resistant to acute loss of DA neurons and gliosis by MPTP.

## Methods

### Ethics, consent and permissions on animals and experimental design

All animal procedures were done in accordance with the guidelines of the Animal Use and Care Committees of Kyoto University (MedKyo08097), Nagoya University (#13151), and Kanazawa University (AP-101606). A line of transgenic mice with a C57BL/6J background harboring the *PrP-Sirt1*cDNA transgene had been generated and deposited at RIKEN Bioresource Center (RBRC06467) as described elsewhere in detail (Watanabe *et al.*, 2014). Mice were reared in a specific pathogen-free environment at 23 ± 2°C, and identified by PCR using a pair of primers, 5′-CAAGAGGTTGTTAATGAAGC-3′ and 5′-TTTCCTGTTGCCTTCAATCAGCTATCG-3′. All comparisons were made between 3–4-month-old, wild-type (WT) and transgenic (Tg) male littermates. Eight mice were subjected to intraperitoneal injection of MPTP (20 μg/g body weight) in saline or saline alone, each 4 times with 2 h-intervals ^17^, followed by histological analysis 4 days later ^3^.

### Histological analysis and quantification

Mice were deeply anesthetized with sodium pentobarbital (50 μg/g, *i.p.*), fixed with transcardial perfusion of 4% paraformaldehyde in 0.1 M phosphate buffer (PB). Frozen-sectioned 10 μm-thick coronal brain sections were reacted with antibodies for tyrosine hydroxylase (TH, rabbit IgG, Chemicon) and glial fibrillary acidic protein (GFAP, mouse IgG, Sigma) ^17, 19^. The sections were reacted with Cy3-conjugated anti-rabbit IgG and FITC-conjugated anti-goat IgG (Jackson ImmunoResearch), and observed with a laser scanning confocal microscope (Eclipse TE2000U, Nikon) with the Nikon EZ-C1 software. We counted TH-positive neurons in the SNpc in three planes (−3.08, −3.16, and −3.40 mm from the bregma), and measured immunofluorescence intensity for TH in the striatum (caudoputamen; CPu) as described previously ^17, 19^.

## Results

Raw data of transgenic supplementation of SIRT1 in a mouse model of MPTP-induced parkinsonism Detailed information can be found in the text file provided (‘Raw data description’). Click here for additional data file. Copyright: © 2015 Kitao Y et al. 2015 Data associated with the article are available under the terms of the Creative Commons Zero "No rights reserved" data waiver (CC0 1.0 Public domain dedication).

We used the original Prp-SIRT1 Tg mouse line that chronically expresses murine *Sirt1*cDNA in the central nervous system (CNS) under control of the murine prion gene promoter ^11, 18^. The expression levels of SIRT1 in the midbrain and striatum assessed by immunoblot were approximately three times higher in heterozygous Tg mice than in the non-Tg (WT) littermates ^12^. Four days after serial administrations of MPTP (20 μg/g body weight), we assessed acute histopathological changes of the nigrostriatal tract in the two genotypes (n = 8).

**Figure 1.**
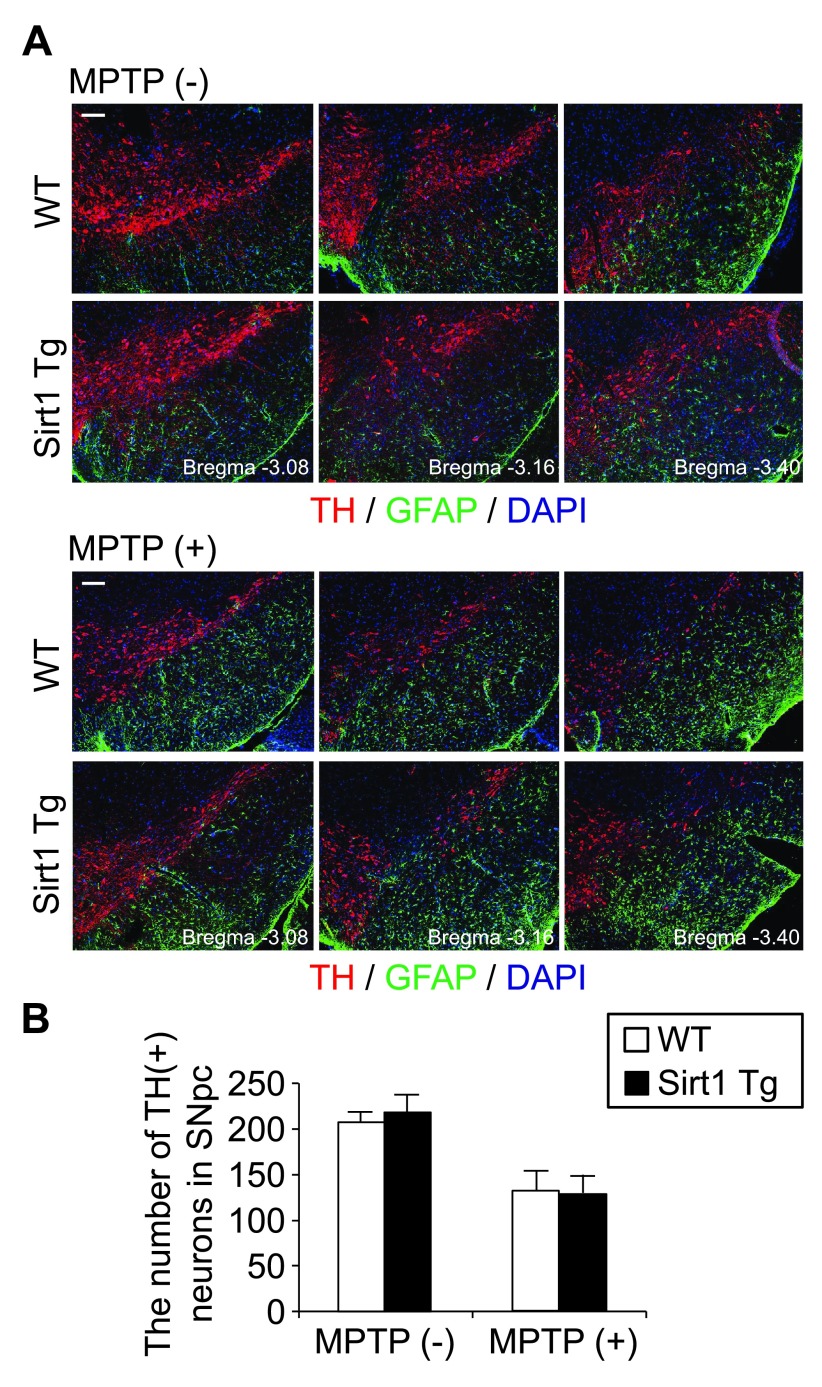
SIRT1 overexpression does not alleviate MPTP-induced loss of midbrain DA neuronal somata and astrogliosis. ( **A**) Representative immunofluorescence images of wild-type and Sirt1Tg mouse midbrain 4 days after intraperitoneal injection of saline with or without MPTP. Serial coronal sections triply labeled for tyrosine hydroxylase (TH), glial fibrillary acidic protein (GFAP), and nuclear DNA (DAPI) consistently exhibited no recognizable histopathological differences in the loss of TH-positive cells (presumed DA neuronal somata and dendrites) and in the proliferation of GFAP-positive astrocytes. Scale bars, 100 μm. ( **B**) The number of TH-positive neurons identified in the three serial coronal sections of SNpc was comparable between the WT and Sirt1Tg littermates. The bars denote mean ± standard error of the mean (n = 4 × 4).

In the midbrain of Tg and WT mice without MPTP administration, the distribution and appearance of TH-positive cells (presumed DA neuronal somata and dendrites), surrounding GFAP-positive astrocytes, and the nuclei of these and other cells (consist mostly of non-DA neurons and microglia) were comparable ( Figure 1A, top). MPTP administration induced acute, significant loss of TH-positive cells and reactive gliosis at comparable severity between the genotypes ( Figure 1A, bottom). The numbers of TH-positive neuronal somata in the SNpc (identified in three serial sections) did not show statistically significant difference ( Figure 1B). These data indicate that the supplementation of SIRT1 does not suppress the loss of DA neurons and reactive gliosis by acute MPTP toxicity.

**Figure 2.**
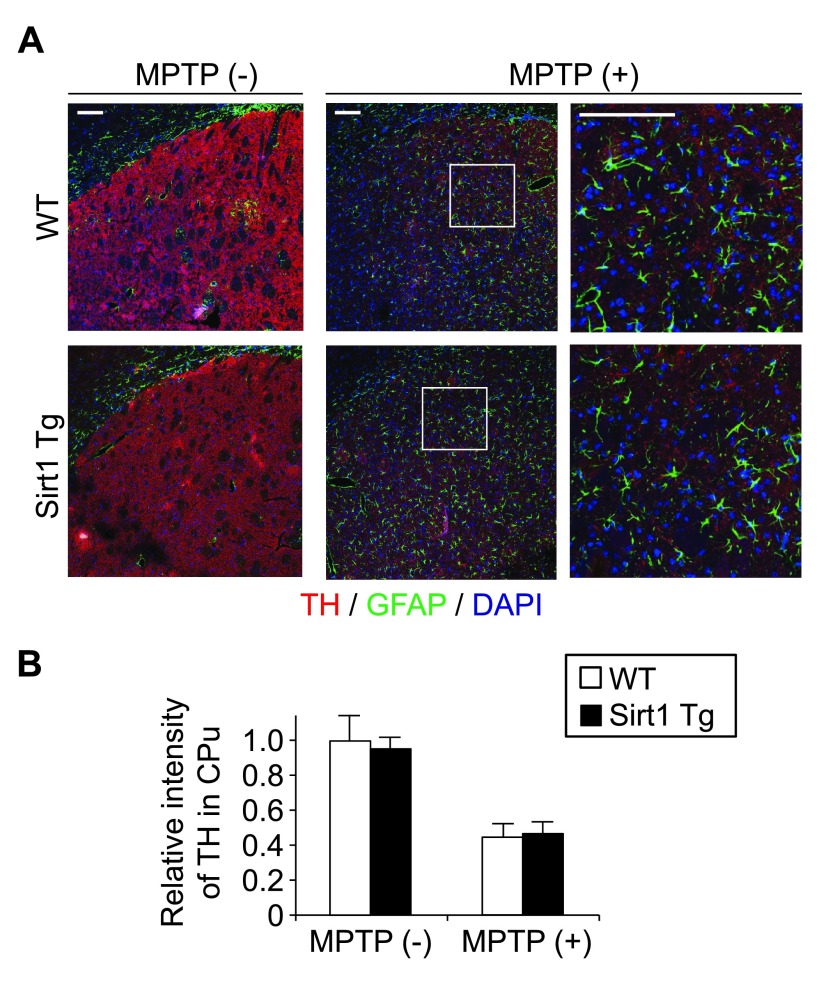
SIRT1 overexpression does not alleviate MPTP-induced loss of striatal DA nerve terminals and astrogliosis. ( **A**) Representative immunofluorescence images of wild-type and Sirt1Tg mouse striatum/caudoputamen 4 days after intraperitoneal injection of saline with or without MPTP. Coronal sections triply labeled for tyrosine hydroxylase (TH), glial fibrillary acidic protein (GFAP), and nuclear DNA (DAPI) exhibited no recognizable histopathological differences in the loss of TH-positive cells (presumed axons and axon terminals of DA neurons) and in the proliferation of GFAP-positive astrocytes. Scale bars, 100 μm. ( **B**) The relative immunofluorescence intensity for TH in the striatum was comparable between WT and Tg littermates. The bars denote mean ± standard error of the mean (n = 4 × 4).

In the striatum/caudoputamen without MPTP administration, the staining patterns for TH (mostly axons and axon terminals of DA neurons), GFAP-positive astrocytes, and the nuclei of these and other cells were comparable between the Tg and WT littermates ( Figure 2A, left). Loss of TH-positive neuropil and reactive gliosis after MPTP administration were also comparable between the genotypes ( Figure 2A, middle; higher magnifications in the right). Fluorescence intensity for TH in the striatum after MPTP administration did not differ ( Figure 2B), indicating that the supplementation of SIRT1 does not alleviate the loss of DA nerve terminals by acute MPTP toxicity.

Overall, PrP-SIRT1 and WT male littermates exhibited similar responses to MPTP toxicity in terms of acute damages to nigrostriatal DA neurons, and proliferation/remodeling of neighboring astrocytes. These findings indicate that supplementation of SIRT1 in neurons and glia does not alleviate MPTP-induced DA neuronal damages and reactive gliosis.

## Discussion

Acute degeneration of DA neurons in the mouse MPTP model can be rescued by resveratrol pre-administration ^4, 5^, or by transgenic supplementation of peroxisome proliferator-activated receptor-γ coactivator 1α (PGC-1α) which controls mitochondrial biogenesis and oxidative phosphorylation ^19^. The rescue effects had been attributed at least partly to SIRT1 ^7, 8^ on the basis that resveratrol directly or indirectly potentiates SIRT1 ^20^, and that SIRT1 activates PGC-1α by deacetylation ^21^. However, transgenic supplementation of SIRT1 either with the neuron-specific promoter ^10^ or with the neuron/glia/vascular endothelial promoter (this study) did not confer tolerance to MPTP-induced pathology. The consistent results indicate that the resveratrol-mediated tolerance to MPTP is due to SIRT1-independent mechanisms ( *e.g.*, antioxidant activity as a polyphenol. See Introduction.), and that SIRT1-mediated activation of PGC-1α is insufficient to confer tolerance ( *i.e*., the upregulation of PGC-1α is necessary). Thus, this study has made a case against the unproven notions that health benefits of resveratrol are attributed mostly to SIRT1, and that potentiation of SIRT1 in neurons and glia nonselectively suppresses neurodegeneration and gliosis. Nevertheless, it is worth testing whether Prp-SIRT1 mice are resistant to chronic neurotoxin models or genetic models of PD ^22, 23^, and whether PGC-1α/SIRT1-double Tg mice are more resistant than the original PGC-1α Tg mice ^19^.

Our recent study with Prp-SIRT1 mice has demonstrated their resistance, albeit limited, to spinal cord degeneration caused by chronic overload of a mutant SOD1 ^11^. The proteotoxic stress by misfolded SOD1 is alleviated at least in part by SIRT1-mediated deacetylation of a master transcription factor HSF1 and the resulting upregulation of HSP70i and perhaps other molecular chaperones ^11^. Intriguingly, either transgenic supplementation of HSP70 or its heat shock-mediated upregulation ( *i.e.*, preconditioning) confers recognizable resistance to MPTP ^13– 15^. We therefore hypothesize that, in DA neurons of Prp-SIRT1 mice, the expression levels of the SIRT1 substrate HSF1 and the downstream effectors including HSP70i are insufficient to counter the toxicity of MPTP―as with the aforementioned situation of PGC-1α. Thus, an obvious subject for future studies is to test whether some preconditioning or milder insults ( *e.g.*, lower dose of MPTP or rotenone) could differentiate Prp-SIRT1 mice from wild-type mice.

## Data availability

The data referenced by this article are under copyright with the following copyright statement: Copyright: © 2015 Kitao Y et al.

Data associated with the article are available under the terms of the Creative Commons Zero "No rights reserved" data waiver (CC0 1.0 Public domain dedication). 

All the original image data are accessible at Kanazawa University Repository, http://dspace.lib.kanazawa-u.ac.jp/dspace/handle/2297/41475?locale=en.

F1000Research: Dataset 1. Raw data of transgenic supplementation of SIRT1 in a mouse model of MPTP-induced parkinsonism. 10.5256/f1000research.6386.d46841 ^24^
