# Supplementary material for: Transgenic supplementation of SIRT1 fails to alleviate acute loss of nigrostriatal dopamine neurons and gliosis in a mouse model of MPTP-induced parkinsonism
Source: F1000Res. 2015 May 27;4:130. [Version 1] doi: 10.12688/f1000research.6386.1 (PMC4482211; doi:10.12688/f1000research.6386.1)
Supplement: Peer review history

# Peer review: Referee response for version 1

**DOI:** 10.5256/f1000research.6849.r8790

**Published:** 2015-09-29

**Authors:** Hiroshi Ichinose

**Affiliations:** 1 Graduate School of Bioscience and Biotechnology, Tokyo Institute of Technology, Yokohama, Japan

The authors examined the possible protective effect of SIRT1 against MPTP-induced neurotoxicity using Prp-SIRT1 transgenic (Tg) mice, which enabled pan-neural over-expression of the SIRT1 gene.  Although the other Tg mice expressing SIRT1 under the promoter of neuron-specific enolase gene had failed to protect MPTP-neurotoxicity previously, this study was worthwhile doing because the Prp-SIRT1 Tg mice had exhibited partial tolerance to some animal models of stroke and neurodegeneration.

In this paper, the authors found no significant difference in MPTP-induced neurotoxicity between the Prp-SIRT1 Tg mice and wild-type by immunohistochemical analyses. Whereas the experimental methods used in this study are sound, immunohistochemical analyses are in general semi-quantitative compared with biochemical ones. As the authors mentioned in the Discussion, it would be interesting to test the milder insults on the Prp-SIRT1 mice. Further studies are expected.

I have read this submission. I believe that I have an appropriate level of expertise to confirm that it is of an acceptable scientific standard.

# Peer review: Referee response for version 1

**DOI:** 10.5256/f1000research.6849.r8871

**Published:** 2015-06-12

**Authors:** Ralitsa I. Petrova

**Affiliations:** 1 Department of Pathology and Cell Biology, Taub Institute for Research on Alzheimer's Disease and the Aging Brain, Columbia University Medical Center, New York, NY, USA

In this study Kitao and colleagues have performed comprehensive *in vivo* analysis of the effect of high levels of SIRT1 on dopamine (DA) neuron survival and gliosis following MPTP insult. The authors test the effect of acute MPTP injury in their previously characterized transgenic mouse line Prp-SIRT1 over-expressing the longevity-related gene *Sirt1* in both neurons and glia. The study reports no alleviation in MPTP-induced neurodegeneration or astrogliosis in Prp-SIRT1 animals compared to wild-type controls. This data is consistent with previously reported inability of SIRT1 gain-of-function to mitigate the effects of MPTP-induced neural damage in a neuron-specific *Sirt1* transgenic line (NSE-SIRT1). Despite the negative connotation of the results, Kitao et. al.’s study is very informative and provides scientifically sound qualitative and quantitative analysis. An important point, also noted by the authors in the discussion, is the possibility that SIRT1 contributes only minimally to DA neuron survival - this effect could be unmasked by using a lower dose of MPTP or alternatively model parkinsonism using the neurotoxin 6-Hydroxydopamine. In either case, further analysis is required to completely negate the neuroprotective effects of SIRT1 in Parkinson's *in vivo* models.

I have read this submission. I believe that I have an appropriate level of expertise to confirm that it is of an acceptable scientific standard.

# Peer review: Referee response for version 1

**DOI:** 10.5256/f1000research.6849.r8792

**Published:** 2015-06-05

**Authors:** Masahiko Takada

**Affiliations:** 1 Systems Neuroscience Section, Primate Research Institute, Kyoto University, Inuyama, Japan

This manuscript deals with the possible alleviative effect of pan-neural supplementation of the longevity-related, pleiotropic deacetylase SIRT1 on a mouse model of MPTP-induced parkinsonism.  The present work was totally based on negative data. However, the entire experimental procedures are sound and the anatomical results obtained are well presented. Since the present work has a potential to provide a certain contribution to the progress in Parkinson’s disease research, this reviewer recommends that the manuscript may be accepted as it is.

I have read this submission. I believe that I have an appropriate level of expertise to confirm that it is of an acceptable scientific standard.
